# A Thought Experiment in Contemporary Drug Development: Informed Bench‐to‐Bedside Strategies

**DOI:** 10.1161/JAHA.112.000031

**Published:** 2013-02-22

**Authors:** Richard C. Becker

**Affiliations:** 1 Duke University School of Medicine, Duke Clinical Research Institute, Durham, NC (R.C.B.)

**Keywords:** Editorials, platelet, thrombosis

## Introduction

The evolution of drug development as a primary objective toward advancing patient well‐being is a fascinating illustration of both scientific progress and reductionism. An advanced understanding of biochemistry, molecular biology, and pharmacology has provided great detail surrounding complex events that govern phenomena in nature, science, and medicine.

In an effort to also understand human disease at a more fundamental level, highly complex components have been reduced to individual parts with the hope that merely “connecting the dots,” small spatial scales, organizational units, and linearity will provide insight and guidance. Traditionally, this approach has led to an understanding of structural–functional relationships and targeted drug therapies for a variety of conditions and diseases. With that being said, in the era of advanced analytical, computational, and technical capabilities, the number of new drug applications (NDAs) and United States Food and Drug Administration (FDA) approvals have declined each year over the past 12 years. Perhaps herein lies a message for the pharmaceutical industry. Perhaps complex systems theories, emergence, and nonlinearity are, in fact, preferred ways to approach the problem. Perhaps “more is different” and the days of reductionism as a means to understanding increasingly complex systems are behind us—committed to posterity.^1^ In this issue of the *JAHA*, Japp and colleagues,^2^ representing an experienced and highly respected group of investigators, provide an illustration of the inherent challenges in applying a target‐based approach to drug development. In this particular case, the target of interest was P‐selectin.

P‐selectin is a platelet α‐granule membrane‐specific protein—one of several hundred proteins either released into the circulation or translocated to the platelet surface following activation. The exposure of P‐selectin is particularly important for platelet–leukocyte interactions and also serves as a 3Cb‐binding protein to initiate complement activation on the platelet surface.^3^ P‐selectin is also present on endothelial cells, where it contributes to endothelial cell–leukocyte interactions. The best‐characterized ligand for P‐selectin is P‐selectin glycoprotein ligand (PSGL)‐1, which can interact with all P‐selectin subtypes under inflammatory conditions.^4^

P‐selectin, as either a marker of platelet activation, prognostic biomarker, or target for inhibition has been an appropriate topic of interest for the past 2 decades.^5–6^ A recombinant soluble immunoglobulin directed against PSGL‐1 showed promise in a porcine model of arterial thrombosis and thrombolysis^7^ and myocardial ischemia–reperfusion injury.^8^ However, 2 phase‐2 clinical trials of patients with acute ST‐segment elevation myocardial infarction (STEMI)^9–10^ failed to show evidence of benefit for improvement of left ventricular ejection fraction, or the occurrence of death, stroke, or recurrent myocardial infarction at 30 or 180 days with administration of a recombinant PSGL‐1 chimera. Do these observations call into question the biology of P‐selectin, PSGL‐1, platelets, leukocytes, or their interaction in the pathobiology of arterial thrombosis or STEMI? Or the translatibility of arterial thrombosis and myocardial ischemia–reperfusion to humans? Or do they underscore the complexity of coagulation, inflammation, and vascular biology from a systems perspective and the inherent limitations of selecting one target among hundreds‐of‐thousands that operate within the complex conditions of human disease?

The development of PSI‐697, an oral P‐selectin antagonist is a direct outgrowth of an academic‐industry partnership employing a meticulous, yet as is often the case, protracted and often expensive approach.^11^ There have been interesting and potentially instructive signals along its evolutionary path. In an inferior vena cava (IVC) stenosis and thrombosis model^12^ PSI‐697 did not reduce either thrombus mass or vessel wall monocyte/total inflammatory cell infiltration, but did reduce vein wall stiffness. In a baboon model of iliac vein thrombosis^13^ PSI‐697 administered beginning 3 days before occlusion and continued for 6 days, did not prevent thrombosis, but did demonstrate greater than 80% vein lumen opening over time and decreased vein wall inflammation compared to low molecular weight heparin and vehicle control. The capability of PSI‐697 to inhibit the binding of human P‐selectin to PSGL‐1, reduce leukocyte rolling, modestly reduce venous thrombosis and thrombus weight, and reduce intimal thickness following arterial injury was demonstrated in vivo and in rodent models of vascular inflammation and thrombosis.^14^ It also reduced ex vivo thrombus formation in a dose‐dependent manner in humans employing the Badimon Flow Chamber.^15^

The current investigation employed a double‐blind, randomized, placebo‐controlled cross‐over design in 24 healthy smokers who received either PSI‐697 600 mg orally or matching placebo. Plasma concentrations of PSI‐697, measured at 4 and 24 hours, increased to 1906 and 83 ng/mL, respectively. The ex vivo addition of thrombin receptor activating peptide (TRAP) increased the concentration of platelet‐monocyte heterotypic aggregates; however, at the plasma concentrations achieved, PSI‐697 did not have a demonstrable effect on either TRAP stimulated or unstimulated platelet–monocyte aggregates. By contrast to P‐selectin binding antibody, PSI‐697 did not inhibit platelet‐monocyte aggregate formation in vitro. There were no safety data reported.

How should these “proof‐of‐concept”‐designed observations be interpreted? Does PSI‐697 exert its primary effect on the vascular surface, preventing leukocyte–endothelial cell interactions? While this may be a contributing mechanism of action, the Badimon Flow Chamber experiments do not fully support this hypothesis. Does P‐selectin, like PSGL‐1, have “Janus faced functions,”^16^ and, under certain conditions, does PSI‐697 *augment* platelet activation and/or platelet–leukocyte interactions? The former was previously proposed in relation to the oral GP IIb/IIIa receptor antagonists and explained by receptor clustering, occupancy, and activation solely if platelets were preactivated and then exposed to a ligand mimetic.^17^ From a practical perspective, should an investment in the compound be made to better understand its mechanism of action for the purpose of further development, and, if so, toward what phenotype or clinical indication? While PSI‐697 may have antithrombotic and/or anti‐inflammatory effects in humans, should a phase‐2 study be performed with clinical endpoints despite a failed proof‐of‐concept study in 24 apparently healthy smokers? The investigators and their sponsor have likely given these questions careful consideration and, after years of good science, close collaboration, and hard work, will need to make a decision.

We currently conduct science and attempt to navigate complex systems using a traditional target‐based approach and relatively primitive tools. Imagine a scenario where drug development begins with a platform derived from a phenotype of interest (informed bench‐work). Now envision a process wherein systems‐based biology is integrated with systems‐based pharmacology and subsequently modeled employing advanced computational clouds. You may now be on the best path toward selecting the right drug for the right condition in the right patient. In the current environment where the tenet of “doing more with less funding” is a well‐recognized reality, “more is indeed different.”
